# Factors Influencing Adherence to Non-Invasive Telemedicine in Heart Failure: A Systematic Review

**DOI:** 10.3390/clinpract15040079

**Published:** 2025-04-10

**Authors:** George Koulaouzidis, Lamprini Tsigkriki, Orestis Grammenos, Sotiria Iliopoulou, Maria Kalaitzoglou, Panagiotis Theodorou, Ioannis Bostanitis, Karolina Skonieczna-Żydecka, Dafni Charisopoulou

**Affiliations:** 1 Department of Biochemical Sciences, Pomeranian Medical University, 70-204 Szczecin, Poland; karzyd@pum.edu.pl; 2 Cardiology Department, General Hospital G. Papanikolaou, 57010 Thessaloniki, Greece; linatsigr@gmail.com (L.T.); orestes1313@hotmail.com (O.G.); sotiria.ili26@gmail.com (S.I.); mariakalaitzo25@gmail.com (M.K.); theod80@hotmail.com (P.T.); bostangiannis@yahoo.gr (I.B.); 3 Paediatric Cardiology Department, Great Ormond Street Hospital, London WC1N 3JH, UK; dafnithess@yahoo.co.uk

**Keywords:** heart failure, telemonitoring, telehealth, telemonitoring, compliance, adherence

## Abstract

**Background/Objectives:** Telemedicine (TM) has emerged as a promising tool for improving heart failure (HF) management by allowing non-invasive, remote patient monitoring. However, patient adherence to TM plays a critical role in its effectiveness. This systematic review aims to assess adherence levels to non-invasive TM interventions and explore factors influencing compliance. **Methods:** This systematic review followed the PRISMA guidelines. A literature search was conducted across the PubMed, Medline, Web of Science, and Google Scholar databases to identify prospective randomized controlled trials published between January 2010 and June 2024. The inclusion criteria included studies focused on non-invasive TM in HF patients with a follow-up period longer than three months. Adherence rates were categorized as high (≥80%), moderate (60–79%), or low (<60%). **Results:** Of the 136 identified studies, 6 met the inclusion criteria. Three studies reported high adherence (>80%), and three moderate adherence (60–79%). Older patients (≥65 years) showed higher adherence, with two studies exceeding 85% adherence. Studies with higher female participation (>30%) reported better adherence, with two exceeding 88%. Across studies, a lack of racial diversity was especially notable, apart from a study that included a population with 69% black and 31% Hispanic participants, where adherence was 50% for ≥10 uploads over a 90-day period. Seasonal variations affected adherence, with December being the lowest (47–69%) and August the highest (>85%). Monitoring multiple health parameters correlated with better adherence (>85%) compared to single-parameter tracking (50–74%). **Conclusions:** TM is a promising tool for HF management, but adherence differs by age, sex, and the complexity of monitoring. To optimize TM use, standardized adherence measures and tailored strategies are needed.

## 1. Introduction

Heart failure (HF) is a significant global health problem, affecting over 64.3 million people worldwide [1,2,3,4]. In the United States, HF leads to approximately 1 million hospitalizations annually, representing 1% to 2% of all hospitalizations [1,2,3,4]. Several primary factors can be attributed to the increasing prevalence of heart failure. Firstly, there has been progress in the management of diseases that raise the probability of developing HF, such as hypertension, diabetes, and myocardial infarction. Furthermore, there are already many medications accessible that can prolong the lifespan of those who have already experienced heart failure. Moreover, the aging of the population in high- and middle-income countries has also played an important role in the increasing burden of HF. This trend is exacerbated by unhealthy lifestyle habits—including poor diet, physical inactivity, and obesity, which increase an individual’s risk for cardiovascular disease and heart failure [1,2]. Recent population data indicate that there has been an improvement in overall survival rates [4]. However, this improvement has been accompanied by a rise in healthcare costs. The main determinant responsible for the substantial costs and notable deterioration in the quality of life of HF patients is frequent hospitalizations. Therefore, there is a significant focus on reducing hospital admissions in the management and treatment of HF [5,6].

The role of telemedicine (TM) in HF management has received considerable attention in recent years, particularly in light of innovative solutions available to enhance patient care. Telehealth refers to technologies that allow for remote monitoring, consultations, and health metrics assessments between patients and healthcare professionals without an in-person visit. This model enables a complete patient assessment, allowing for timely changes to medical therapy. Importantly, studies showed that telemedicine may lead to reduced hospitalization rates for HF and significant clinical improvements [7,8,9,10,11,12,13,14,15,16,17,18]. TM serves as a valuable tool for ongoing patient education, promoting self-care and improving adherence to treatment [10]. This can leave patients feeling more in control and having closer ties to their healthcare team [11,12]. TM also increases access to healthcare, addressing geographic disparities [14,16,18]. These disparities are often due to the unequal distribution of healthcare resources, with rural areas generally having limited access to specialist care, diagnostic services, and timely follow-ups. That is where telemedicine comes in, as it can help bridge that gap by giving patients the ability to receive monitoring and expertise in a continuous fashion without excessive travel [19,20,21,22,23,24].

Non-invasive TM involves patient participation in a daily auto-evaluation routine, which is transmitted to a care facility [25,26,27]. The HF team regularly examines the transmitted data, looking for trends over extended periods of time, or alternatively received alerts if any variable falls outside a preset limit [28,29,30,31]. If early indicators of cardiac instability are detected, then a therapeutic response is triggered [32,33,34,35]. A meta-analysis showed that non-invasive TM significantly reduces the risk of all-cause mortality and HF hospitalization in HF patients [36].

However, non-invasive telemonitoring systems are limited by the fact that they need patients to strictly follow instructions. While wearables and Bluetooth technologies offer convenience, they do not eliminate the necessity of adherence to the technology itself [37,38,39,40].

This systematic review aims to assess adherence levels to non-invasive TM interventions and explore factors influencing compliance.

## 2. Materials and Methods

### 2.1. Protocol

This systematic review was conducted according to the Preferred Reporting Items for Systematic Reviews and Meta-Analyses (PRISMA) guidelines [41]. The protocol was prospectively registered with the International Prospective Register of Systematic Reviews and has been allocated the registration number CRD42024563922 (www.crd.york.ac.uk/prospero, accessed on 16 July 2025).

### 2.2. Search Strategy

A literature search was conducted to identify studies that reported compliance with telemonitoring in HF patients. Two reviewers independently screened titles and abstracts in the PubMed, Medline, Web of Science, and Google Scholar databases in August 2024 to identify eligible studies published between January 2010 and June 2024. Discrepancies between the two reviewers were resolved by discussion or through the involvement of up to two further reviewers. The initial reviewers were selected based on their clinical and research experience in cardiology and digital health, ensuring familiarity with both heart failure and telemedicine interventions. The additional reviewers were included to provide methodological oversight and maintain consistency with the predefined inclusion criteria. All the reviewers independently assessed the studies to minimize bias. Other limits included studies published in English. The keywords or MeSH (Medical Subject Headings) used during the search were ‘telemedicine’ OR ‘telehealth’ OR ‘telemonitoring’ OR ‘remote monitoring’ OR’ home monitoring’ AND ‘heart failure’ AND ‘compliance’ OR ‘adherence’ OR ‘acceptance’. The references of eligible studies and published systematic reviews were also searched to identify any additional studies.

### 2.3. Inclusion and Exclusion Criteria

Studies were eligible if they reported compliance or adherence outcomes with home non-invasive telemonitoring in HF patients. Only prospective, randomized control trials (RCTs) were considered. Furthermore, the included studies were required to employ home-based non-invasive TM service and to have an interventional study design. Heart failure was defined according to the criteria applied in each included study. The majority of studies used a combination of clinical parameters such as left ventricular ejection fraction (LVEF ≤ 45%), New York Heart Association (NYHA) functional classification (typically class II–IV), and/or a history of hospitalization due to HF within the preceding 12 months.

### 2.4. Data Extraction

Two authors independently extracted the data from the included studies, verified by a third author AT. The extracted data are study name, first author, year of publication, country of origin, study type, monocentric or multicentric study, number of subjects, percentage of male population, duration of follow-up, inclusion criteria and exclusion criteria, and description of TM intervention. This information, along with the compliance rates, was organized in tables to provide a summary of the studies.

The primary evaluated outcome was patient adherence to non-invasive telemonitoring protocols. Adherence was defined based on the criteria specified in each individual study. To standardize the analysis across studies, we categorized adherence rates into three groups: high adherence (≥80%), moderate adherence (60–79%), and low adherence (<60%), in alignment with the previous literature [42,43]. Definitions varied slightly among studies—for example, some measured adherence as the number of days with transmitted data over the expected monitoring period, while others used the number of uploads or compliance with specific parameters.

### 2.5. Risk of Bias Assessment

Two independent reviewers assessed the risk of bias using the revised Cochrane risk-of-bias tool for randomized controlled trials (ROB-2) [44]. RoB2 assessed 5 domains: randomization process, effect of assignment to intervention, missing outcome data, measurement of the outcome, and selection of the reported result. Overall quality was assessed as low risk (“low risk” in all domains), some concerns (at least 1 domain rated “some concern”), and high risk (at least 1 do-main rated “high risk” or 2 to 3 domains rated “some concerns”). The evaluation was independently evaluated by 2 reviewers. Discrepancies among the reviewers were resolved through discussion and consensus.

## 3. Results

**Figure 1:**
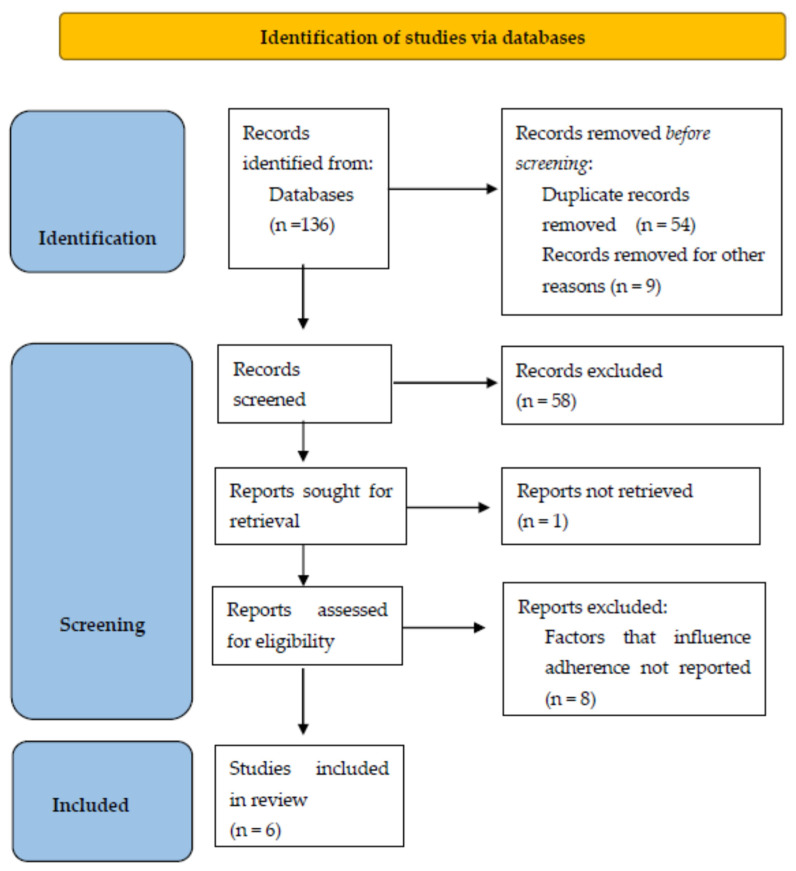
PRISMA flow diagram of the literature screening process.

The initial search produced 136 results. Following the elimination of duplicate articles, a total of 73 articles were included in the title and abstract screening process. Among these, 14 articles seemed relevant, and we performed a full-text review/evaluation, resulting in a total of 6 articles being eligible and included in this systematic review [45,46,47,48,49,50]. The detailed selection process is illustrated as a PRISMA flow diagram in Figure 1.

### 3.1. Risk of Bias

The quality of studies was found to be high. Details are provided in Table 1.

### 3.2. Studies Characteristics

The included studies were conducted in a wide range of countries, including the United States (two studies), Europe (two studies), Asia (one study), and Australia (one study). An overview of the results is provided in Table 2 and Table 3. The largest study included 1571 participants, while the smallest study cohort comprised 104 participants. The shortest duration of follow-up was 3 months and the longest was 18 months. All six studies asked the patients to measure their weight. The BP was asked to be measured in three studies, the HR in three studies, the oxygen saturation rate in one, and the body composition in one. Finally, questions regarding health status were used in two studies.

In the OSICAT trial, the majority of the patients had a more advanced stage of the disease. Specifically, 71% of the patients were recruited while they were in the hospital, 2.6% of the patients received a heart transplant during the study, and 10.0% of the patients were classified as NYHA class IV. Similarly, 10% of the participants in the BEAT-HF study were NYHA class IV, while those with lower socioeconomic status represented 39.4% of the cohort. On the contrary, in the TIM-HF2 study, less than 1% of the patients were in NYHA class IV, while patients with serious depression were not included in the study. In the study by Pekmezaris et al., the participants were non-White, and in more detail: 31% were Hispanic and 69% were black. In the same study, the vast majority of the patients (72%) had low socioeconomic status. In the Homes-HF study, only patients with NYHA class II and III were included. Patients with severe depression or dementia and access to a telephone line were excluded. Finally, in the ITEC-CHF a high proportion of patients were suffering from chronic diseases, while patients with severe cognitive impairments were excluded.

### 3.3. Adherence

To summarize the reported compliance in this analysis, three studies were classified as reporting high compliance [45,49,50] and three studies as reporting medium compliance [46,47,48]. Nevertheless, differences were seen among the studies over the precise definition of ‘compliant’ and ‘noncompliant’. For example, Pekmezaris et al. defined as high adherence the transmission of 10 or more uploads over 90 days [50]. Conversely, Ding et al. adhered to the Australian clinical recommendations for managing HF, which describe good compliance as uploading weight measurements at least 6 days per week [48,51].

### 3.4. Age

In five out of the six included studies, the mean or median age of the HF patients was ≥65 years [45,46,47,48,49], whereas in just one study it was <65 years [50]. Within the trials that included individuals aged 65 years and older, two studies have shown a high level of adherence [45,49], whereas three studies indicated a moderate level of adherence [46,47,48]. In the study conducted with those below the age of 65, a high degree of adherence was seen [50].

### 3.5. Sex

Male participants represented the vast majority in all six studies. Out of the three trials where the female participants accounted for ≤30% of the entire group [45,46,48], compliance was rated as medium in two of them [46,48] and as high in one of them [45]. Conversely, in the three remaining trials where the female composition ranges from >30% to <40% of the group, compliance rates were high in two studies [49,50] and medium in one [47].

### 3.6. Race

There is only one study that looked exclusively at black and Hispanic patients, and it reported a high level of compliance [50]. But it is worth mentioning that in this study, adherence was defined as 10 or more uploads over 90 days. Another study revealed a high representation of these populations as well, but it did not elaborate on the compliance [47].

### 3.7. Place of Residence

Variations in telemonitoring compliance based on place of residence were examined in one study [45]. Patients living in rural areas showed higher compliance compared with patients living in urban areas.

### 3.8. Follow-Up Period

Throughout the research period, there were no observed changes in the degree of compliance (Table 4). Compliance was consistently high in two studies [45,49] and moderate in two others [46,47] throughout the length of the trials. In two trials, no relevant data were provided [48,50]. The participants showed a reduced probability of sending measurements on weekends as compared to weekdays [47]. Adherence varied by month, with lower levels observed in the winter months. December showed the lowest adherence across all months, while August had the highest amount [47].

### 3.9. Number of Recorded Parameters

The number of sent parameters did not seem to hinder the level of compliance. Three studies that assessed the participants’ weight transmission demonstrated a modest level of compliance [46,47,48]. Conversely, three studies that required the participants to send several parameters demonstrated a high level of compliance [45,49,50].

## 4. Discussion

The present review highlights several challenges in patient compliance and adherence, which directly impact the effectiveness of TM interventions. One of the key takeaways from the review is the significant variation in adherence across different studies. Three studies reported high adherence, while three reported moderate adherence. The variability in adherence rates may be attributed to differences in how compliance was defined in the respective studies. For instance, in the study by Pekmezaris et al., high adherence was defined as 10 or more uploads over 90 days [50], while Ding et al. followed the Australian clinical guidelines [51], defining high adherence as uploading weight measurements at least six days per week [48]. However, such definitions are highly dependent on the specific characteristics of a given study population. This clearly highlights the need for standardized definitions that are clinically relevant and not designed only for the specific setting of a trial.

Patient characteristics, such as age and sex, were shown to affect adherence to TM. For example, most studies included older patients (≥65 years), and adherence was generally higher among older adults. This finding aligns with previous research suggesting that older patients may be more motivated to adhere to TM interventions as a means of managing their chronic condition and avoiding hospitalization [52,53,54]. Also, older persons frequently maintain stronger ties to caregivers which may bolster adherence by offering reminders, encouragement, or assistance with technology utilization. For those with cognitive impairment or reduced functional capacity, such assistance is essential for facilitating consistent engagement in telemonitoring practices.

Furthermore, gender disparities were observed, with female patients representing a smaller proportion of the study participants. The review indicates that in trials where female participation was low, adherence rates were typically moderate, whereas higher female representation correlated with higher adherence. This suggests that gender may play a role in TM compliance, possibly due to differences in health-seeking behaviors between men and women [55]. Moreover, many women might not be aware of telemedicine services, leading to reduced participation in studies. That can create a lack of gender diversity, leading to skewed findings that do not fully address the experience of female patients [56]. That, in turn, can perpetuate health disparities because research results may not generalize as well to women’s health problems [57]. Finally, the underrepresentation of women may explain some of the negative associations between female gender and telemedicine use in some studies [58].

A significant concern is the comparatively lower level of representation of persons of non-White race in the research. Among the six studies included in this review, only one study specifically examined the black and Hispanic populations with HF. This is an enduring issue. During their analysis, Granger et al. found that just four studies assessed the use of technology for self-management in the black population [59].

Interestingly, the review found no significant changes in adherence over time, suggesting that patients who initially engaged with TM interventions generally maintained their compliance throughout the study period. This contrasts with the trend in adherence to medication, which tends to decline over time for reasons such as treatment fatigue, adverse effects, or waning perceived benefit. One possible explanation is that telemonitoring—unlike medication intake—is a more interactive experience, offering patients immediate feedback, a greater amount of contact with their care teams, and a sense of empowerment and engagement that may help maintain motivation.

However, seasonal variations were observed, with adherence declining during winter months, particularly in December, and peaking in August. These seasonal trends may be related to changes in patients’ routines, such as holidays or weather conditions, which could affect their ability to engage with TM technology consistently. Moreover, caregiver and family support may be reduced during holiday time, resulting in less adherence for those who depend on others to perform monitoring activities.

Another notable factor influencing adherence was the complexity of the TM interventions. Studies that required patients to monitor multiple parameters generally reported higher adherence rates than those focusing solely on single measurements, such as body weight. This finding suggests that patients may be more engaged when they are asked to participate in more comprehensive monitoring activities, perhaps because they perceive the intervention as more robust and beneficial [60].

One study [45] evaluated differences in telemonitoring adherence by place of residence. Rural patients had better adherence compared to urban patients. Telemonitoring may be a unique opportunity for rural patients to regularly and in real-time connect with their healthcare providers. By contrast, patients who live in urban areas, despite having better access to health services, are likely to perceive less added value in telemonitoring, which may explain their lower engagement level with telemonitoring. These findings underscore the potential of telemedicine to close care gaps in underserved geographic areas and suggest that patient context should guide the design of remote care programs.

Another notable issue is the increased incidence of rejection during the recruitment phase. In the two major trials, TIM-HF2 and BEAT-HF, the proportion of subjects who declined the offer to participate in the trial accounts approximately for 50% and 33% of the cohort study, respectively. This is consistent with the findings published by Gorst et al. in their systematic study [61]. This is an existing problem and the proportion of patients who refuse TM is largely unknown. Research is needed to quantify the rates of patient uptake, refusal, and abandonment of telehealth, to understand the number of patients who are willing to accept and use it. Research also needs to explore patient beliefs and perceptions about telehealth to try and explain why patients decide to take up, refuse, abandon, or sustain their use of telehealth.

### 4.1. Standardizing Terminology

Defining and measuring compliance in-home telemedicine is highly important for improving patients’ healthcare efficiency, achieving system interoperability, and exploring research progress. The primary effort is to create a common language by ensuring that terms like “compliance” and “adherence” are well defined in order to avoid confusion. Also, a standardized compliance level can be a helpful approach (for example: compliant, partially compliant, or noncompliant). In order to carve out consistency, both the objective and subjective metrics should be standardized. Also, we can improve uniformity of assessments by defining compliance measurement intervals (e.g., daily, weekly, or monthly).

### 4.2. Improving Compliance

Patient engagement is the core of success for any healthcare model, and more so in telemedicine care. Many strategies have been proposed to improve patients’ adherence to telemedicine care [62,63]:

The Benefits Must be Clear: Acceptance and participation improve by providing information on how telemedicine can improve their health outcomes.

Patient-Centered Education: The designs should be patient-centered and consider the literacy levels of different populations and technological familiarity factors.

Make Technology User-Friendly: The availability of enough technical support by healthcare professionals can also improve the patients’ capability of using these services effectively.

Personalized Telemedicine Plans: Considering that adherence might differ among various populations, customized interventions should be introduced to address the requirements of different patient cohorts.

Strong Patient–Provider Relationships: Ongoing follow-ups, seamless availability of medical representatives to answer the patient questions, and encouragement for the involvement of caregivers or family members can drastically enhance adherence and patient experience.

## 5. Study Limitations

A major limitation of this review is the variability in adherence definitions and measurements in the studies that were included, which makes it difficult to directly compare. Also, no separate analysis based on HF categories was performed in any of the included studies. Furthermore, the review predominantly encompasses studies that are undertaken in high-income settings, which limits the applicability of its findings to low-resource contexts. Additional limitation arises from the underrepresentation of some demographic groups, especially non-White ethnic minorities and low socioeconomic individuals, which could affect the generalizability of the results.

## 6. Conclusions

In conclusion, while non-invasive TM has the potential to improve heart failure management, patient adherence remains a critical determinant of its success. The findings of this review underscore the importance of addressing barriers to compliance, including socioeconomic factors, patient education, and the complexity of TM interventions. Further research is needed to develop strategies for enhancing adherence and optimizing the effectiveness of TM programs in diverse patient populations. Standardizing adherence metrics across studies would also allow for more accurate comparisons and a clearer understanding of the factors that influence patient engagement with TM.

## Figures and Tables

**Table 1:** Results of quality appraisal.

Study	D1	D2	D3	D4	D5	Overall
TIM-HF2 [45]	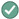	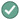	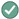	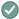	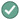	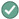
OSICAT [46]	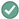	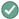	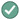	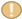	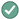	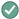
BEAT-HF [47]	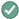	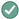	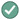	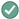	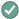	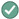
ITEC-CHF [48]	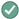	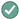	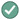	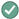	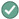	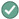
HOMES-HF [49]	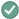	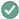	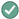	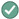	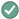	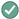
Pekmezaris et al. [50]	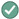	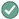	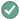	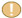	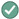	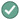

**Domains:** D1: randomization process; D2: deviations from the intended interventions; Domain D3: missing outcome data; D4: measurement of the outcome; D5: selection of the reported result. **Judgment:** 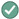 low risk; 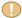 some concerns.

**Table 2:** Overview of the studies included in the systematic review, with a focus on the study protocols. RCT: randomized controlled trial; TM: telemedicine; UC: usual care; HFpEF: heart failure with preserved ejection fraction; HFmrEF: heart failure with mildly reduced ejection fraction; HFrEF: heart failure with reduced ejection fraction.

Study	Study Type	Country/Centers	Duration	Sample Size	Age (Years)	Males (%)	Ethnicity (%)	HF Categories
TIM-HF2 [45]	Prospective, RCT (1:1)	Germany; 200	12 months	1571 (796 TM, 775 UC)	70 ± 10	70	n/a	HFpEF 25% HFmEF 30% HFrEF 45%
OSICAT [46]	Prospective, RCT (1:1)	France; 13	18 months	990 (507 TM, 483 UC)	69.9 ± 12.4	72.3	n/a	HFpEF 21.7% HFmEF 19.8% HFrEF 58.5%
BEAT-HF [47]	Prospective, RCT (1:1)	USA; 6	6 months	1437 (722 TM, 715 UC)	70.9 ± 14.1	53.8	White 50.7 Black 23.2 Hispanic 13.2	EF (mean) 42.7
ITEC-CHF [48]	Prospective, RCT (1:1)	Australia; 2	6 months	184 (91 TM, 93 UC)	70.1 ± 12.3	76.6	n/a	HFrEF 100%
HOMES-HF [49]	Prospective, RCT (1:1)	Japan; 27	12 months	181 (90 TM, 91 UC)	67.1 ± 12.8	57	Japanese 100	EF (mean ± SD) 40.5 ± 11.4
Pekmezaris et al. [50]	Prospective, RCT (1:1)	USA; 1	90 days	104 (46 TM, 58 UC)	59.9 ± 15.1	59	Black 69 Hispanic 31	HFpEF 29% HFmEF 10% HFrEF 61%

**Table 3:** Overview of the studies included in the systematic review with a focus on the technical approaches. LVEF: left ventricular ejection fraction; HF: heart failure; BP: blood pressure; HR: heart rate; NYHA: New York Heart Association (NYHA).

Study	Inclusion Criteria	Data Transmitted	Intervention	Adherence
TIM-HF2 [45]	LVEF ≤ 45%, or if >45% treated with oral diuretics; NYHA II or III; inpatient for HF within 12 last months	Weight, BP, ECG, and self-rated health status	Physician-led medical support for 24/7	The ratio between the number of days with measurements performed and the number of days with measurements possible
OSICAT [46]	Inpatient for HF within 12 months ago	Weight and eight symptom questions	Structured telephone support and nurse-led collaborative care	The ratio of the number of days with body weight measurement divided by the effective days *
BEAT-HF [47]	Older adults being inpatients for HF during recruitment	Weight	Structured telephone support	Count of adherence days from 0 (no transmission) to 7 (daily transmission) in event-free weeks
ITEC-CHF [48]	EF ≤ 40%	Weight	Structured telephone support and nurse-led collaborative care	Monitoring days per/180 days × 7 days/week
HOMES-HF [49]	NYHA II–III; admission for HF within 30 days of enrolment	BP, HR, weight, and body composition	Nurse-led collaborative care	Monitoring days/ days that measurements should be performed in a month × 100%
Pekmezaris et al. [50]	NYHA I–III	BP, oxygen saturation rate, weight, and HR.	Structured telephone support and nurse-led collaborative care	Low: <10 uploads over 90 days High: ≥ 10 uploads over 90 days

* effective days: number of days in the study minus the number of days when weight could not be measured.

**Table 4:** Reported compliance results throughout the study period.

		ADHERENCE			
	3 Months	6 Months	9 Months	12 Months	18 Months
TIM-HF2 [45]	>85%	>85%	>85%	>85%	
OSICAT [46]	50.4 ± 31.4	74 ± 35.3	69.8 ± 36	68.8 ± 36.8	65.7 ± 37.6
BEAT-HF [47]	69%	53.3%	-	-	-
ITEC-CHF [48]	-	97% with ≥4 uploads/ week	-	-	-
HOMES-HF [49]	96.2%	90.4%	88.5%	90.9%	-
Pekmezaris et al. [50]	50% with <10 uploads	-	-	-	-

## Data Availability

No new data were generated or analyzed in this study. Data sharing is not applicable to this article as it is based on a systematic review of the publicly available literature.
